# Substance use and the HIV care continuum: important advances

**DOI:** 10.1186/s13722-018-0114-4

**Published:** 2018-03-12

**Authors:** P. Todd Korthuis, E. Jennifer Edelman

**Affiliations:** 1 Department of Medicine, Section of Addiction Medicine, Oregon Health and Science University, 3181 SW Sam Jackson Park Rd., L475, Portland, OR 97239-3088 USA; 2 Yale University School of Medicine, New Haven, CT USA

The HIV care continuum identifies five stages of HIV medical care: (1) HIV diagnosis, (2) linkage to HIV care, (3) engagement and retention in HIV care, (4) treatment with antiretroviral therapy (ART), and (5) achievement of HIV viral suppression—all of which are essential to effectively treat and prevent the spread of HIV. Most recent estimates indicate that in the United States, 85% of people living with HIV are diagnosed, 62% are linked to care, and only 49% achieve HIV viral suppression [1]. People who use drugs and alcohol are particularly likely to experience gaps in each stage of the HIV care continuum. While antiretroviral therapy has dramatically improved the life expectancy for people living with HIV, important disparities remain among people with substance use that threaten individual and public health [2]. Screening for and treating substance use may close these gaps in outcomes and help achieve the UNAIDS 90-90-90 goal of 90% diagnosis, 90% ART treatment, and 90% HIV viral suppression by 2020 [3].

Addiction Science and Clinical Practice’s special thematic series, “Substance Use and the HIV Care Continuum,” sponsored by the National Institute on Drug Abuse (NIDA) Clinical Trials Network, focuses on publications that advance understanding of how identifying and treating substance use contributes to the HIV care continuum in U.S. and international settings. The series includes original research on the adverse effects of alcohol, drugs and structural barriers on the HIV care continuum, novel strategies for improving engagement in HIV treatment, and protocols for three recently implemented major clinical trials to improve engagement and retention in HIV care. The authors adhere to recent recommendations for using less stigmatizing, patient-centered terms when referring to substance use [4, 5].

While progress has been made toward the UNAIDS 90-90-90 goals, challenges remain. In an observational study of people using drugs in Oakland, California, Lambdin et al. [6], reported that criminal justice involvement was associated with increased rates of HIV and hepatitis C testing but did not result in improved treatment engagement. Those who reported receiving substance use disorder treatment, however, were more likely to engage in HIV care than those not receiving treatment. In Pretoria, South Africa, women who reported recent physical assault were more likely to be newly diagnosed with HIV, and those using opiates or cocaine were less likely to receive antiretroviral therapy [7]. Similarly, female entertainment and sex workers in Thailand who used methamphetamines were less likely to be retained in care at 12 months [8].

Alcohol is often overlooked as a potentially detrimental contributor to adverse HIV outcomes. Cook et al. [9] carefully examined the role of alcohol, demonstrating that people with HIV who report heavy drinking experienced increased odds of failing to achieve sustained HIV viral suppression.

Systems issues must also be addressed to close HIV care continuum gaps for people who use drugs. Qualitative interviews with public health and HIV service providers in Connecticut identified the need to increase mental health and substance use services and peer navigation/case management services and the need to decrease service agency stigma as key barriers and potential facilitators for improving HIV testing, engagement, and treatment [10]. Idrisov et al. [11] reported no association between individual injection use histories and HIV outcomes, suggesting that systems issues may also prove a dominating contributor to HIV outcomes in Russia, obscuring individual factors.

The special series highlights several areas of progress toward interventions to overcome such barriers. Contingency management, the strategy of rewarding people for desired behaviors, was a key component of Project HOPE, a NIDA Clinical Trials Network study that used peer navigators to link hospitalized patients with untreated HIV and substance use disorders to HIV treatment. Providing monetary incentives to attend peer navigator sessions increased visit attendance and HIV viral suppression at 6 months [12]. Similar strategies might be tested in outpatient HIV clinics where the prevalence of substance use disorders remains high.

Integration of HIV care and addiction treatment might also improve HIV outcomes. Mburu et al. [13] used a peer-led community-based approach to increase HIV testing and treatment services in Cambodia. Simeone et al. [14] found 93% retention and HIV viral suppression among patients receiving integrated methadone and HIV primary care in an opioid treatment program—much higher than when HIV primary care was delivered off-site. Similarly, in-depth interviews with HIV providers and patients in Tanzania endorsed a preference for integrating HIV care in methadone treatment settings [15]. Additional implementation and comparative effectiveness studies are urgently needed to increase understanding of optimal approaches for integrating HIV and substance use treatment, both in clinical and non-clinical settings (e.g. AIDS service organizations). Integrating treatment approaches such as buprenorphine into general healthcare settings help to change a medical culture that has long stigmatized people with substance use disorders in the U.S. and other countries [16].

Novel study protocols and a feasibility trial published in the special series report the design of promising interventions to improve HIV treatment and engagement. Westergaard et al. [17] reported that a peer navigation intervention with a smartphone application to aid in care coordination was acceptable and feasible to HIV-infected patients for improving visit attendance and ART adherence. Other trials aim to address systems improvement interventions. Claborn et al. [18] describes a randomized trial of a tablet-based mobile platform to improve care coordination between HIV and substance use providers. Garner et al. [19] describe protocols for two multi-site, cluster randomized trials: one testing the Addiction Technology Transfer Center change quality improvement strategy with or without an organizational change intervention in AIDS service organizations, and a second trial of motivational interviewing training for AIDS service organization staff versus usual care [20]. Both trials study intervention effects on client treatment engagement and HIV outcomes.

Taken together, these publications advance the science of interventions to improve engagement in the HIV care continuum for people with substance use disorders and point to the need for additional research. Many community-based settings with high HIV prevalence, such as some substance use disorder treatment facilities and office-based buprenorphine providers, still do not routinely test for HIV [21]. Implementation trials to promote testing in these settings would likely increase the proportion of people living with HIV who know their diagnosis. Substance use in rural settings continues to grow, with risk of HIV and hepatitis C outbreaks in areas with limited treatment resources. Studies to engage HIV testing and treatment retention among people using drugs in rural settings are urgently needed. Correctional settings also offer opportunities to initiate treatment for substance use and relapse prevention interventions that increase the likelihood of engaging in HIV treatment and achieving HIV viral suppression.

Closing gaps in the HIV care continuum requires the development and testing of new interventions directed at people who use drugs, and improved implementation strategies for the interventions that are already known to work. As the HIV epidemic matures, it provides an important framework for translating health improvements for other chronic conditions, such as treatment of substance use disorders and chronic hepatitis C. Lessons learned from interventions to improve engagement in the HIV care continuum across diverse settings may serve as models to address the care continuum for these and other conditions.
